# Medical and Surgical Society of Baltimore, Md.

**Published:** 1878-02

**Authors:** John J. Caldwell


### MEDICAL AND SURGICAL SOCIETY OF BALTIMORE, MD.

### *Remarks following Prof. A. B. Arnold's Address on Hydrophobia.*

BY JOHN J. CALDWELL, M. D.

Mr. President:—The few remarks we shall make, following the exhaustive observations of the learned Professor upon this mysterious and almost unknown disease, will have for their object to call the attention of the society to.

First. The terminology.

Second. The analogy existing between this and other incubative poisons.

Third. To some points of treatment.

Fourth. To refer to a few cases under our own observation.

It would be well if the Profession could adopt another name which would have a better and closer relation to the pathology and symptoms of this malady. As "toxæmia canina vel felina," or the poisonous effects following the bite of rabid dogs or cats.

This poison has its inception, its incubation, and its explosion, or final demonstration, as well as its point or points of election.

The same may be said of the bites of venomous serpents or insects, which either act locally or overwhelm the nervous system; also of the poison of small pox and scarlet fever, which select the surfaces; of puerperal fever, electing the peritoneum; of typhoid poisoning, electing the mucous membrane of the bowels; or spotted fever, electing the meninges of the brain and spinal cord. Thus the poison commonly known as rabies virus, no matter where inserted, elects for its point of explosion the brain, the upper portion of the spinal cord, the muscles of the neck and chest; while strychnine and tetanus, choose the spinal axis, leaving the brain free. In hydrophobia the brain, the medulla, and the cervical spine are, in the last stages, more or less hyperæmic; hence the proneness to frequent clonic spasm, and the irritation of light, of sound, of draughts of air, of particles of fluids, or solids, upon the muscles of deglutition. Indeed, so hyperæsthetic are these centers that the sligtest irritation will produce convulsion. The pathology of this disease is involved in great obscurity; indeed, the same may be said of all incubating poisons acting by reflex forces upon distant centers, in consequence of minuteness of nerve vesicles, requiring the aid of powerful microscopic observation to witness these minute changes, as well as the spectrum to appreciate the chemical and vital metamorphosis. None of our savans have been able to trace the "materies morbi," or its pathological tracts from the point of inception to their field of explosion in any of these incubative poisons. But what particuarly maintains with the poison under discussion; what especially distinguishes it from the others, is its election of three of the most vital points of the economy, viz: the brain apendages, the respiratory and circulatory centers, while other incubating and elective diseases involve more remote and less vital localities. Here, we believe, rests the explanation of the great dread of, and fearful mortality from, hydrophobia, of tetanus, and poison of strychnia, but especially with the first named, for the brain, and its membranes and the upper part of the spinal cord, and sometimes the nerves of the wound, the mouth, the windpipe, and the lungs, are found in a congested state, no doubt principally from the loss of function, and thus we can account for the unhappy symptoms of pain, and uneasiness in the stomach, throat, and lungs, during the last scene or stage of the disease, for the stiff and unwieldy tongue, for the indistinct speech, for the rigid mucles of the throat, for the impeded deglutition, the dread of swallowing, and the convulsions of the muscles of this locality, and for the irregular mental disturbances. The causes have been generally attributed to rabid cats and dogs, but later writers have attributed the disease to several other wild and domestic animals, while laboring under the various passions, such as anger, rage, fright, heat, etc. A late writer has claimed the disease uniformly resulting from the bite of a pole-cat or skunk. In an article upon the subject (we think in the Virginia *Medical Monthly*, for September) is given a large experience and much data from field of practice of the writer in Nevada. In our own experience of four cases (three while a student in Bellevue, and one in private practice), one that proved rapidly fatal was caused by the bite of a cat while the pound master was endeavoring to rescue her from a number of dogs in the pound.

Again, we read, from the *Evening News*, of a remarkable case of hydrophobia:

Chicago, Sept. 16.—A singular case of death from hydrophobia occurred here last Saturday. The facts of the case have just been made public, and are as follows: Charles Haake, a boy six years of age, was bitten on the head by a cat, which he was chastizing for some reason, about four weeks ago. The wound was so slight that the child did not complain of it at all, but on Saturday morning last, he was taken with all the symptons of hydrophobia, and died before night. The cat has never at any time exhibited any signs of madness, and the physicians who attended the boy regard it as the most remarkable case extant.

TREATMENT.

As to prophylaxis. It is the uniform and established plan, by most practitioners, to cauterize and wash the wound as soon as praticable, the sooner the better. Indeed, if this mode should maintain throughout the non-professional as well as the professional world, many valuable lives might be saved. We would suggest that this, as well as many other medical truths, should be universally taught and practiced, as is the case among the barbaric and semi-barbaric races.

We will here cite a case occurring some ten or twelve years ago, wherein a little girl was badly bitten by a rabid dog. It so happened, while visiting one of the suburban villages of New York City, her grandfather sent her to the village Post office with a pony-cart and driver. While waiting at the store door, a dog came running along snapping at everything in his way. He bit her pony through the nose. During the struggle she was thrown to the ground, the dog biting her through the pectoral muscles of the right side, incising, lacerating, scratching and bruising the parts in a terrible manner. A few minutes after this accident we were by her side. We washed the parts thoroughly and cauterized them with nitro-muriatic acid. Our patient recovered, and at our last hearing was doing well, enjoying the best of health (several years after the accident), while the pony and other animals bitten by this dog suffered and died with this disease within a few weeks.

INCUBATION.

If during the stage of incubation an agent could be introduced by the mouth or rectum which would have the power of acting as an antidote, and thus neutralize the poision which is circulating in the blood, and prevent its acting perniciously upon the nervous centers and especially the brain and medulla spinalis, the discoverer would confer a boon upon humanity and place himself in the temple of fame side by side with the immortal Jenner.

REMEDIES.

Of all remedies, none are so fully adapted to the symptoms as chloroform, chloral hydrate and the croton-chloral hydrate; to allay and lessen the hyperæmia and hyperæsthesia of the neural tripod of life, the brain, medulla spinals and great sympathetic, in order to maintain life's currents and facilitate the molecular change, and thus eradicate the poison, the constant galvanic current should be exhibited powerfully and continously through these implicated axes. If necessary, they should be continued for any number of days. Nourishment should be adminstered by the rectum and hypodermically.

In conclusion, we will cite, the following to corrobrate our theory.

Hammond, page 550: " Dr. Schward, of Milan, attempted the cure of this disease by the primary galvanic current. In one case the current was feeble, and was continued for nineteen hours. Great improvement ensued, the oppression disappeared, and the dysphagia was entirely relieved. Through some misunderstanding advantage was not taken of these ameliorations, and the patient was allowed to die. In the other case, which was one of undoubted hydrophobia, occurring in a girl nine years old, the current from twenty two Daniel's cells was employed. The current was passed from the soles of the feet to the forehead, for fifty-eight hours, almost contincusly, and the duration of the disease prolonged to seven days and seven hours, when the patient died. During the last two days there were no hydrophobic symptoms.

